# Betalain-Rich Concentrate Supplementation Improves Exercise Performance in Competitive Runners

**DOI:** 10.3390/sports4030040

**Published:** 2016-07-25

**Authors:** Justin S. Van Hoorebeke, Casey O. Trias, Brian A. Davis, Christina F. Lozada, Gretchen A. Casazza

**Affiliations:** 1 Sports Performance Laboratory, University of California Davis Sports Medicine Program, 3301 C St., Suite 1600, Sacramento, CA 95816, USA; jsvanhoorebeke@ucdavis.edu (J.S.V.H.); cotrias@ucdavis.edu (C.O.T.); cflozada@ucdavis.edu (C.F.L.); 2 Physical Medicine and Rehabilitation, University of California, Davis, Medical Center, 4860 Y St., Suite 1700, Sacramento, CA 95817, USA; badavis@ucdavis.edu

**Keywords:** beetroot, time trial, muscle damage markers, rate of perceived exertion

## Abstract

This study aimed to determine the effects of a betalain-rich concentrate (BRC) of red beets, containing antioxidant and anti-inflammatory properties, on performance and exercise-related muscle damage. Thirteen (25.3 ± 5.4 years) competitive male runners completed two double-blind, cross-over, randomized trials (BRC and control) separated by seven days. Each trial was preceded by six days of supplementation with 100 mg of BRC or control. On the seventh day, exercise trials commenced 150 min after supplementation with 50 mg BRC or control and consisted of 30 min of treadmill running (77 ± 4% VO_2_max) followed by a 5-km time trial (TT). During exercise at the same intensity, BRC resulted in a 3% lower heart rate, a 15% lower rate of perceived exertion (RPE) and a 14% lower blood lactate concentration compared to the control (*p* = 0.05). Five-kilometer TT duration (23.0 ± 4.2 versus 23.6 ± 4.0 min) was faster in 10 of the 13 subjects, and RPE was lower (*p* < 0.05) with the BRC treatment compared to the control. Lactate dehydrogenase, a marker of muscle damage, increased less from baseline to immediately and 30 min after the 5-km TT with the BRC treatment, despite no differences in subjective measures of muscle soreness and fatigue. In summary, BRC supplementation improved 5-km performance time in male competitive runners.

## 1. Introduction

Beetroot has received much attention from competitive athletes, coaches and scientists as a natural food supplement for improving exercise performance [1,2]. Several studies have found a correlation between nitrate-rich beetroot juice (5–9 mmol of nitrate or about 85 g of beets) and 5–16.1-km time-trial performances in competitive runners and cyclists [3,4,5,6]. The nitrate in the beetroot juice was thought to increase the production of nitric oxide through the nitrate-nitrite-nitric oxide reduction pathway induced by bacterial nitrate reductases in the oral cavity [1,2]. Higher nitric oxide could improve muscle blood flow and oxygenation and, thus, exercise time trial performance.

Beetroot also contains a high concentration of betalains, highly bioactive phytochemicals [7] lauded for their free radical scavenging capacity [8] and protecting cell membranes from lipid peroxidation and heme decomposition [9]. This property is likely due to the phenol and cyclic amine groups in betalains, which are good electron and proton donors [9]. The ability of betalains to neutralize superoxide radicals may also lead to an increase of nitric oxide availability in the blood and subsequently increase blood flow and oxygen delivery. Betalains have also been shown to express potent anti-inflammatory properties by reducing the pro-inflammatory cytokines tumor necrosis factor alpha (TNF-α) and interleukin-6 in patients with osteoarthritis [10]. Since intense exercise has been shown to increase muscle damage, inflammation and oxidative stress [11], betalain supplementation may be able to reduce that oxidative stress and inflammation and result in improved exercise performance.

Although data supports beetroot juice intake and improved exercise performance, research investigating the health and exercise performance benefits of betalains is limited. To our knowledge, no study has investigated the effects of a nitrate- and sugar-depleted, betalain-rich concentrate (BRC) on exercise performance. Therefore, the primary aim of this study was to examine the effects of BRC on 5-km time trial time in competitive, male runners, while secondary outcomes included assessing BRC effects on muscle damage, muscle soreness and overall fatigue. We hypothesized that BRC supplementation would improve exercise performance by decreasing muscle damage and subjective measures of muscle soreness and fatigue compared to the control.

## 2. Materials and Methods

### 2.1. Subjects

We recruited 15 recreationally-competitive male runners (25.3 ± 5.4 years; 173.5 ± 4.9 cm; 70.6 ± 7.9 kg) from the University of California at Davis campus and local venues to participate in the study. Twelve subjects were needed based on a power analysis [12] (power = 0.8, significance *p* = 0.05, mean difference (MD) = 1.0 min for performance time of supplement versus water and SD of the MD = 1.1 min) [13]. Two subjects were excluded due to noncompliance, leaving 13 subjects for data analysis. Participants had to be healthy, nonsmokers and run more than 8 miles per week. Written informed consent was obtained as approved by the Institutional Review Board of the University of California at Davis.

### 2.2. Screening and Baseline Measures

Day 0 consisted of medical-clearance and measurements of height in cm using a stadiometer, body mass in kg using a scale and body composition via 7-site skinfolds using a Harpenden caliper [14]. Subjects completed a maximal treadmill test (Stairmaster Clubtrack at 1% slope) to determine work intensities for the experimental trials. Every 2 min of the test, oxygen consumption (VO_2_max; TrueOne 2400, ParvoMedics, Sandy, UT, USA), heart rate (HR) in beats per minute (Model 5410, Polar, Woodbury, NY, USA) and rate of perceived exertion (RPE) (0–10-point scale) [15] were measured. Exercise began at 8–11 kph and increased by 0.8 kph every 2 min to exhaustion. The metabolic cart was calibrated prior to each trial at various flow rates (50–400 L/min) and with a standard gas mixture of 16% O_2_ and 4% CO_2_.

### 2.3. Experimental Trials

Through a double-blind, randomized and counterbalanced method, subjects were assigned BRC (Racerunner^®^, FutureCeuticals, Momence, IL: serving size: 1 capsule (50 mg beetroot concentrate), 5 kcal, 0.1 mg protein, 1 mg carbohydrate, 0 mg fat, 0.3 mg fiber and 12.5 mg betalains) or control (serving size: 1 capsule, oat ß-glucans, Nutrim^®^, FutureCeuticals, Momence, IL: 19 kcal, 1 mg protein, 3 mg carbohydrate, 0.4 mg fat and 0.9 mg fiber). Oat ß-glucans were used as a control to match as close as possible all contents other than the betalains in the experimental treatment. Treatments were supplied inside a blue and white pill capsule and were identical in appearance, taste and smell. Six subjects started with the betalain treatment, and seven started with the control treatment. Prior to each trial, subjects supplemented with 50 mg of treatment, twice per day (30 min before breakfast and 30 min before dinner) for 6 days, recorded 7 days of training (training log; type, duration, intensity and miles of training) and logged 3 days of their diet (Day 5–7) (MyFitnessPal, Inc., San Francisco, CA, USA). We chose 50 mg, as that was shown to be the minimal effective dose in a study by Pietrzkowski et al., 2010 [10]. Diet and exercise were followed exactly prior to the second trial. Subjects reported to the lab between 8:30 and 10:00 a.m. in a fasted state. Baseline muscle soreness and fatigue were recorded with 100-mm visual analogue scales (VAS) from “no pain” to “extreme pain” and from “not tired” to “utterly exhausted” [13]. Baseline blood was sampled via a 22-gauge forearm vein catheter, and VO_2_, the respiratory exchange ratio (RER) and HR were measured. Following baseline measurements, 50 mg of BRC or control were given with 7 mL/kg of water. Subjects rested for 140 min before commencing exercise to allow BRC to reach peak concentrations in the blood [7]. Thirty minutes post-supplementation, subjects consumed a snack (Smuckers’ Uncrustables^®^, Strawberry, 210 kcal: 56% CHO, 22% fat, and 22% protein; The T.M. Smucker Company, Orrville, OH, USA) with 3 mL/kg of water to prevent hypoglycemia and to simulate pre-training/competition behavior. At 140 min, subjects completed a 10-min warm up, voided their bladder and had body mass measured.

### 2.4. Submaximal Exercise

Treadmill exercise commenced 150 min post-supplementation. Speed was adjusted to elicit 75% of VO_2_max during the first 15 min. After 15 min, subjects straddled the treadmill while blood was drawn; 3 mL/kg of water were consumed, and they were connected to the metabolic cart. HR and RPE were averaged over the last 10 min of exercise when the steady state had been reached. A blood sample was collected immediately after the 30-min submaximal exercise period, and 3 mL/kg of water were provided. Blood values were reported as an average of the 15- and 30-min time points. The same workload and rest periods were used for the second trial.

### 2.5. Time Trial

Following the submaximal exercise bout, subjects completed a 5-km time trial (TT), where the subjects controlled their speed and had access to their distance, but were blinded to their actual speed and HR. Elapsed time, RPE and HR were recorded every 1.67 km and then reported as an average over the entire 5-km time period. Post TT, blood was immediately collected, and 3 mL/kg of water were ingested while subjects completed 5 min of active recovery at 4.8 kph.

### 2.6. Post-Exercise

Lastly, 30 min after the 5-km TT, VO_2_, RER and HR and body mass were recorded; a blood sample was collected, and VAS scales were completed. Before leaving, subjects ingested 50 mg of supplement with 3 mL/kg of water to maximize BRC’s effects on recovery [7]. Twenty four hours post-exercise, VAS scales were recorded followed by a 24-h blood draw.

### 2.7. Blood Analysis

Standard serum biochemistry analyses for lactate dehydrogenase (LDH), creatine kinase (CK) and glucose were performed on collected blood utilizing a Piccolo Xpress Chemistry Analyzer (Abaxis, Union City, CA, USA). Blood lactate was determined with the Lactate Plus analyzer (Nova Biomedical, Waltham, MA, USA), and hematocrit was measured using microhematocrit tubes (Statspin, Norwood, MA, USA). Serum samples were stored at −80 °C prior to analysis.

### 2.8. Statistical Analysis

**Figure 1:**
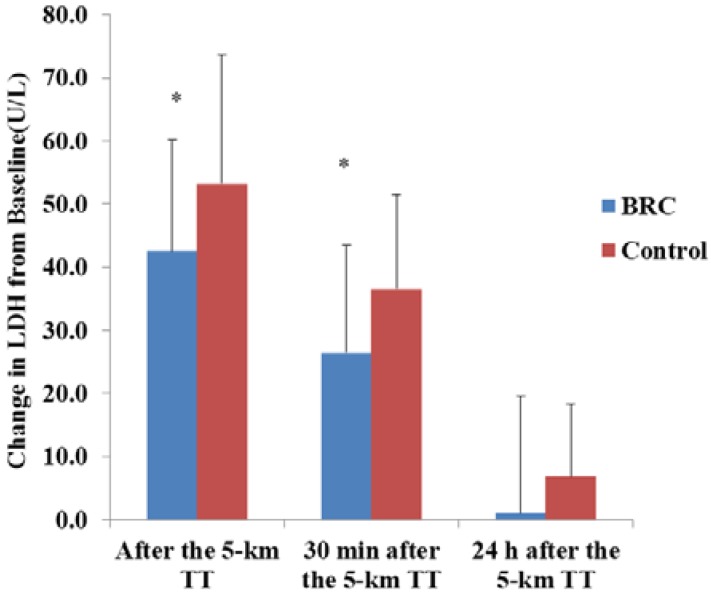
Change in serum lactate dehydrogenase (LDH) from baseline to immediately after the 5-km time trial (TT), baseline to 30 min after the 5-km TT and baseline to 24 h after the 5-km TT. *n* = 13 men; mean ± SD. * Different from the control, *p* ≤ 0.05. BRC, betalain-rich concentrate.

Data are presented as the means ± standard deviation (SD). The normality of the distribution for each variable was tested using the Shapiro–Wilk test. All variables except time to complete the 5-km TT were normally distributed (*p* > 0.05). Paired *t*-tests were used for baseline, submaximal exercise and time trial comparisons of HR, VO_2_, RER, blood lactate, serum glucose, serum LDH and CK, as well as baseline measures of whole body muscle soreness and fatigue. Paired tests were also used for change values for LDH (Figure 1) and CK. The time trial data were not normally distributed, and a nonparametric analysis was used with the Wilcoxon signed rank test (StatView software, Version 5.0.1, SAS Institute Inc., Cary, NC, USA). Significance was accepted at *p* ≤ 0.05.

## 3. Results

### 3.1. Subjects

Physical characteristics of the subjects are presented in Table 1. The average daily amount of calories consumed and macronutrient proportions from the 3-day diet records were 2388 ± 617 kcal, 45 ± 8% carbohydrate, 21 ± 5% fat and 34 ± 7% protein for BRC and 2361 ± 608 kcal, 44 ± 7% carbohydrate, 21 ± 5% fat and 35 ± 6% protein for control. Weekly training volumes were identical between treatments at 24.8 ± 10.2 miles, 6.2 ± 3.4 h and at an average RPE of 5.4 ± 1.6.

### 3.2. Baseline Measures

Baseline HR, VO_2_ and RER were similar between treatments. There were no treatment differences in baseline blood lactate, serum glucose serum CK and whole body muscle soreness and fatigue. Baseline LDH was higher with the BRC treatment (144.6 ± 26.7 versus 136.0 ± 16.8 U/L; *p* = 0.04).

### 3.3. Physiological Responses to Submaximal Exercise

Submaximal exercise values are reported in Table 2. Speed and % VO_2_max were similar between treatments. We found no significant differences in VO_2_ or RER; however, HR was 2.7 ± 0.8%, and RPE was 14.8 ± 1.3% lower with BRC. Six subjects had a lower average HR with Trial 1 (two control and four BRC), and seven had a lower average HR for Trial 2 (two control and five BRC). Five subjects had a lower average RPE with Trial 1 (two control and three BRC), and eight had a lower average RPE for Trial 2 (two control and six BRC).

There were no differences in serum glucose, lactate dehydrogenase or creatine kinase between treatments, but blood lactate was 13.8 ± 1.6% lower with BRC treatment compared to the control with the submaximal exercise bout (Table 2). Four subjects had a lower average lactate with Trial 1 (one control and three BRC), and nine had lower average lactate for Trial 2 (three control and six BRC).

### 3.4. Time Trial

Data from the 5-km TT are presented in Table 3. BRC supplementation was associated with a 3.0 ± 1.9% increase in 5-km speed and a 6.8 ± 1.0% decrease in RPE. There was also a 36-s reduction in 5-km TT time with BRC supplementation. Ten of the 13 subjects had improved TT times with BRC compared to the control. Eight subjects had a faster 5-km TT for their second trial (three with the control and five with BRC), and five subjects had a faster TT for Trial 1 (one control and four BRC).

The changes in LDH from baseline to after the 5-km TT were 25.1 ± 1.2% (*p* = 0.001) lower with BRC treatment (Figure 1). There was no difference in the change in serum CK, whole body muscle soreness and whole body fatigue from baseline to after the 5-km TT. Hematocrit increased more from baseline to immediately post the 5-km TT with BRC treatment (2.9 ± 1.3%) compared to the control (1.3 ± 2.1%) (*p* = 0.004). However, subjects were well hydrated during the trials, as body weight changes were minimal and not different between treatments (−0.76 ± 0.3 kg and −0.73 ± 0.3 kg for BRC and the control respectively; *p* = 0.75).

### 3.5. Physiological Responses during Recovery

Heart rate was 86 ± 9 and 91 ± 10 bpm (*p* = 0.0002) 30 min after the 5-km TT for the BRC and control condition, respectively. LDH changes from baseline to 30 min after the 5-km TT were 38 ± 1.3% lower (*p* = 0.05) with the BRC treatment (Figure 1). Serum glucose was 5.3 ± 0.5% higher (*p* = 0.05) 30 min after the 5-km TT in the BRC treatment compared to the control. There were no differences in whole body muscle soreness and fatigue or serum CK or LDH 24 h after the 5-km TT. However, while both CK and LDH remained elevated above baseline (*p* = 0.05) with the control treatment 24 h after the 5-km TT, both values had returned to baseline 24 h after the 5-km TT with the BRC treatment.

## 4. Discussion

Competitive athletes explore nutraceuticals as a way to improve exercise performance and recovery. One such compound is betalain-rich concentrate, an extract of beetroots [7]. This study aimed to assess the effects of the only commercially-available betalain supplement, which contained 12.5 mg of betalains on exercise performance. The principal finding of this investigation was that supplementation with 50 mg of BRC, which did not contain nitrates or sugars, 2.5 h prior to exercise and after six days of pre-loading, decreased 5-km running time in 10 of the 13 young, healthy, male runners. In addition, blood markers of muscle damage (serum CK and LDH) increased with exercise for both treatments, but returned to baseline 24 h post-exercise only with BRC supplementation. Serum LDH increased to a lesser extent from baseline to after the 5-km TT and baseline to 30 min after the 5-km TT with BRC supplementation. 

### 4.1. Muscle Damage

As a dietary compound, functionally integrated into a class with polyphenols, betalains have anti-inflammatory and anti-oxidant properties [16], which are thought to aid in reducing reactive oxygen species and cellular damage produced during times of stress [17]. This is further supported by the fact that reduction in the inflammatory phase response has been shown to improve exercise performance and hasten recovery [18]. Dietary antioxidants, such as vitamin C and E, carotenoids, flavonoids and phenolic compounds, have been shown to counteract the cellular damage associated with exercise [19,20]. We know of only one human study to examine the effects of betalains on inflammation, and that study found that 35, 70 and 100 mg of betalain supplementation resulted in a significant reduction in inflammatory cytokines—TNF alpha, IL-6, GRO-alpha and RANTES—which translated into a dose-dependent reduction in pain (McGill test) in osteoarthritic subjects [10]. Yet, to our knowledge, no study has tested whether or not these functions of betalains translate into reduced exercise-related muscle damage in healthy, young athletes. Our study found that BRC treatment blunted exercise-related muscle damage, as shown by smaller increases in the muscle damage marker LDH. 

### 4.2. Physiological Responses to Exercise

Although we could find no studies examining the effects of betalains on the physiological response to exercise, there are several studies that have examined the effects of beetroot juice. When examining the oxygen cost of submaximal exercise at the same intensity with 0.5 L of beetroot juice (BRJ) (5–7 mmol of nitrate and 85 g of beets) in trained, but not elite athletes, most of the studies have shown a decreased VO_2_, but no differences in HR, RER or blood glucose and lactate [1,2,21]. The lower oxygen consumption was thought to be related to increased plasma nitrite and nitric oxide (NO) production from the nitrates in the BRJ, increasing blood flow and oxygenation to type II muscle fibers [1]. The BRC supplement from our study did not contain nitrates, so it was not surprising that we saw no treatment difference in VO_2_. However, although the differences were small, we did see an overall significant effect of HR, blood lactate and RPE due to a lower level in nine of the 13 subjects with BRC supplementation during submaximal exercise at the same exercise intensity. The mechanism for the reduced HR, blood lactate and RPE is unknown and most likely occurred through a different mechanism than improved blood flow from increased plasma nitrates, as seen in BRJ supplementation. It has been suggested that NO-availability can be reduced by superoxide radicals [22]. Therefore, it is possible that the BRCs anti-oxidant properties may have improved NO-induced vasodilation by maintaining NO availability. However, this is just speculation, as we did not measure plasma NO. Although evidence exists showing the vasodilatory effects of antioxidants, such as vitamins C, E and α-lipoic acid [23], we know of no study that has shown these effects with betalains, and thus, more research is warranted. 

### 4.3. Five-Kilometer Time Trial Performance

In our study, BRC treatment led to a significant reduction in muscle damage as indicated by lower LDH levels and greater resistance to fatigue as supported by lower RPE values. This suggests that BRCs may have provided resistance to cellular damage, which ultimately translated to faster speeds and a significant improvement in the 5-km time trial in 10 out of 13 subjects. While we know of no human studies that have examined exercise-related muscle damage and betalains, a study that injected betalains intraperitoneally showed a strong anti-inflammatory effect on carrageenan-induced paw edema and peritonitis in mice, as measured by reduced production of superoxide anion and the cytokines TNFα and IL-1β, as well as increased anti-inflammatory IL-10 levels (17).

While we know of no studies examining the effects of betalains on exercise performance, there have been several studies showing improved exercise performance with beetroot juice (BRJ) supplementation [3,4,5]. Lansley et al. (2011) used a randomized, double-blinded, cross-over study design and found that one dose of BRJ (6.2 mmol nitrates) given 2.75 h prior to exercise was associated with an ~2.8% decrease in both 4-km and 16.1-km TT cycling time in competitive cyclists compared to a nitrate-depleted BRJ that served as a control (*p* < 0.01) [4]. Cermak et al. (2012) also used competitive cyclists and found that six days of supplementation with BRJ (8 mmol nitrates) improved 10-km TT performance compared to a nitrate-depleted BRJ [3]. The improved performance in these studies was thought to be related to the nitrates in the BRJ. Our BRC supplement did not contain nitrates, but we still saw a performance benefit in our runners. Since both the nitrate-containing BRJ and the nitrate-depleted BRJ contained betalains, it is possible that the performance benefits of the nitrate-containing BRJ may have been even greater if a placebo had been used as a control instead. The BRJ serving sizes (0.5 L or 85 g of beets or the equivalent of approximately 102 mg of betalains) in the aforementioned studies were considerably higher than the BRC dosage (~13 mg of betalains) in our study, which may explain the larger performance differences seen in the BRJ studies.

### 4.4. Post-Exercise Recovery Period

BRC supplementation resulted in no differences in the serum muscle CK levels (a marker of muscle cell damage) compared to the control. However, there was a significant reduction in lactate dehydrogenase (LDH) levels from baseline to immediately after and 30 min after the 5-km TT. While serum CK and LDH were still significantly elevated 24 h after exercise with the control treatment compared to baseline, they were not significantly different at 24 h after the 5-km TT with BRC supplementation. We hypothesized that since a reduction in the inflammatory phase response has been shown to improve exercise performance and hasten recovery [19], BRC supplementation may have blunted inflammation and muscle damage and, thus, conferred improvements in exercise performance. However, while we did see improved exercise performance, there were no significant differences in the post-exercise subjective measures of muscle soreness and fatigue between treatments.

### 4.5. Limitations and Future Directions

A limitation of this study may have resulted from too low of a betalain dose (~13 mg) compared to concentrations found in whole beets (~100 mg). The use of oat ß-glucans as a control may have limited the effects the BRC, since it does have some immunostimulatory properties. Future studies should use a more inert substance as the control. We may have found larger improvements in performance and greater reductions in muscle damage had we used a larger dose. Furthermore, the type of exercise chosen (75% sub maximal running exercise and 5-km TT) may not have been long enough or intense enough to cause significant muscle damage in these athletes. Although our subjects were well trained, they were not elite runners, and we chose the 5-km TT, as that is a typical racing or training distance of many recreational runners. We most likely would have seen a more robust response if we used eccentric exercise of higher intensity and duration, but that is not what recreational runners typically do and is less applicable to a real-life situation. Exercise that involves more eccentric muscle contractions should be evaluated in future studies. Measurements of blood pressure and nitric oxide may have shed light on potential mechanisms for our findings, while measurements of serum BRC would have improved the validity. Thus, further studies should aim to incorporate higher concentrations of BRC and eccentric exercises to improve upon the validity of this study.

## 5. Conclusions

Low-dose BRC supplementation, 2.5 h prior to exercise, resulted in significantly lower submaximal HR, RPE and blood lactate at the same exercise intensity. These advantages translated to an increase in speed and a reduction in the subsequent 5-km trial time and RPE, potentially facilitated by the protective effect conferred by betalain-rich concentrate. Lastly, BRC supplementation facilitated quicker post-exercise recovery. Therefore, given the lower HR, blood lactate, RPE, muscle damage and improved 5-km TT performance, we conclude that BRC supplementation can improve exercise performance and recovery in healthy, young competitive runners.

## Figures and Tables

**Table 1:** Subject physical characteristics; *n* = 13 men; VO_2_, oxygen consumption.

Variable	mean ± SD
Age, y	25.3 ± 5.4
Height, cm	173.5 ± 4.9
Weight, kg	70.6 ± 7.9
Body fat, %	9.6 ± 2.3
Fat-free mass, kg	63.7 ± 6.6
Fat mass, kg	6.8 ± 2.1
VO_2_max, mL·kg^−1^·min^−1^	55.6 ± 3.7
Training hours per week	6.0 ± 3.3
Running km per week	39.8 ± 3.3

**Table 2:** Physiological responses with the 30-min submaximal exercise bout; *n* = 13 men; VO_2_, oxygen consumption; LDH, lactate dehydrogenase. * Significantly different from the control. BRC, betalain-rich concentrate.

Variable	BRC	Control	*p*-Value
Average speed, kph	12.2 ± 0.7	12.2 ± 0.7	1.0
Heart rate, bpm	165.0 ± 11.2 *	169.5 ± 10.7	0.04
VO_2_, L·min^−1^	3.01 ± 0.39	3.01 ± 0.33	0.99
% VO_2_max	76.9 ± 4.4	77.0 ± 3.7	0.88
Respiratory exchange ratio	0.92 ± 0.04	0.93 ± 0.04	0.09
% energy from carbohydrate	71.1 ± 15.3	76.6 ± 13.6	0.09
% energy from fat	28.9 ±15.3	23.4 ± 13.6	0.09
Rate of perceived exertion	3.79 ± 1.47 *	4.35 ± 1.28	0.04
Blood lactate, mmol·L^−1^	2.9 ± 1.6 *	3.3 ± 1.4	0.05
Serum glucose, mmol·L^−1^	5.0 ± 0.4	5.0 ± 0.5	0.70
Serum creatine kinase, U·L^−1^	379.9 ± 242.4	369.3 ± 225.7	0.75
Serum LDH, U·L^−1^	173.6 ± 23.5	169.2 ± 27.2	0.49

**Table 3:** Physiological responses with the 5-km time trial (TT); *n* = 13 men. LDH, lactate dehydrogenase; * significantly different from the control. BRC, betalain-rich concentrate.

Variable	BRC	Control	*p-*Value
Average speed, kph	13.3 ± 1.9 *	12.9 ± 1.8	0.04
Time to complete the TT, min	23.0 ± 4.2 *	23.6 ± 4.0	0.04
Average heart rate, bpm	176.0 ± 14.5	178.3 ± 13.3	0.31
Rate of perceived exertion	5.9 ± 1.1 *	6.3 ± 1.0	0.03
Blood lactate, mmol·L^−1^	6.7 ± 4.1	6.4 ± 3.2	0.36
Serum glucose, mmol·L^−1^	5.6 ± 1.6	5.5 ± 1.1	0.59
Serum creatine kinase, U·L^−1^	427.3 ± 267.3	424.7 ± 250.7	0.94
Serum LDH, U·L^−1^	187.2 ± 30.4	189.2 ± 32.5	0.67
